# Supplementary material for: A systematic approach for quantitative orientation and phase fraction analysis of thin films through grazing-incidence X-ray diffraction
Source: J Appl Crystallogr. 2025 Jul 22;58(Pt 4):1288–98. doi: 10.1107/S1600576725004935 (PMC12321010; doi:10.1107/S1600576725004935)
Supplement: Supplementary file 1 [file j-58-01288-sup1.pdf]

JOURNAL OF APPLIED CRYSTALLOGRAPHY

Volume 58 (2025)

Supporting information for article:

A systematic approach for quantitative orientation and phase fraction analysis of thin films through grazing incidence X-ray diffraction

Fabian Gasser, Sanjay John, Jorid Smets, Josef Simbrunner, Mario Fratschko, Victor Rubio-Gimenez, Rob Ameloot, Hans-Georg Steinruck and Roland Resel

S1. Calculated peak positions and intensities

There exists a variety of literature on the determination of Bragg peak positions in reciprocal space (Shmueli, 2006; Simbrunner et al., 2018). For this work, the software GIDVis (Schrode et al., 2019) was used to calculate Bragg peak positions as well as the structure factors F_{hkl} and peak multiplicities m_{hkl} required in eq. 4. An example of the calculated peak positions and squared structure factors $|F_{hkl}|^2$ of the metal-organic framework ZIF-8 (cubic with $a = 16.991$ Å (Park *et al.*, 2006)) with uniplanar texture and (001) contact plane is shown in Fig. S1a. The individual Bragg peak positions can be transformed into spherical reciprocal space coordinates, yielding q_{hkl} and ψ_{hkl}, as visualized in Fig. S1b. To calculate accurate integrated intensities, peak multiplicities m_{hkl} have to be taken into account. Peak multiplicities occur due to the overlap of equivalent Bragg peaks as a result of the cylindrical symmetry inherent in a uniplanar texture or the spherical symmetry in a powder texture. Fig. S1 clearly shows how multiple Bragg peaks contribute to the same Debye-Scherrer ring, emphasizing the importance of using appropriate multiplicity factors when comparing different textures.

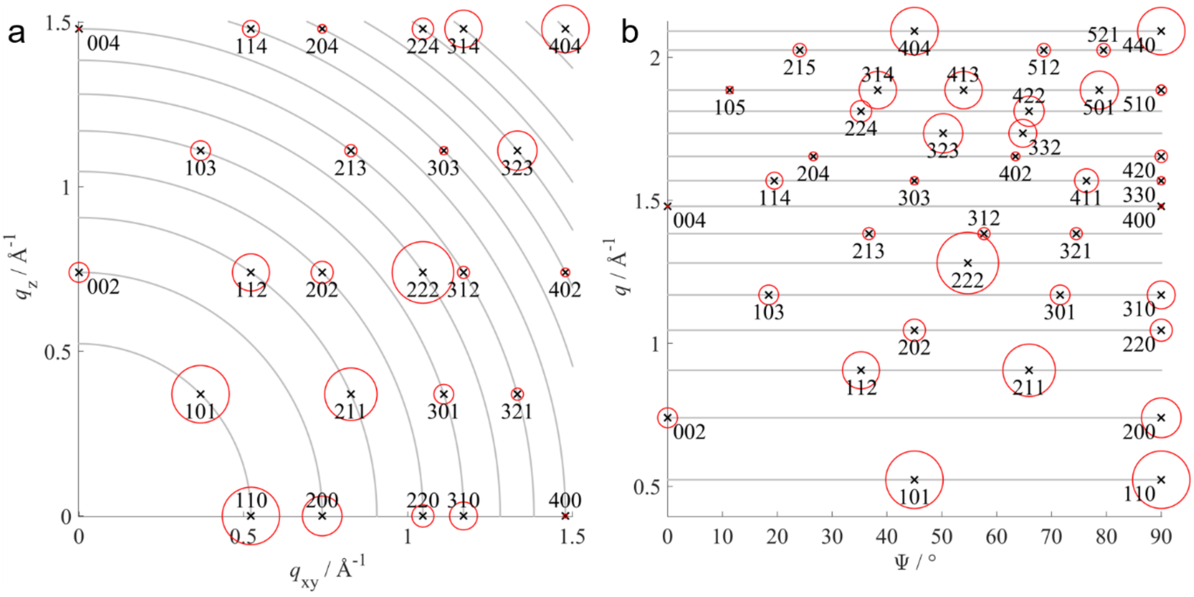

Figure S1 Calculated peak positions and intensities of ZIF-8 visualized in (a) cylindrical reciprocal space coordinates and (b) spherical reciprocal space coordinates. The black crosses correspond to the positions of Bragg peaks for ZIF-8 crystallites with uniplanar texture, where the (001) crystallographic plane is parallel to the substrate. The area of the red circles corresponds to the squared structure factors of the individual peaks multiplied with respective peak multiplicities. The grey lines show the Debye-Scherrer rings obtained for a ZIF-8 powder sample.

S2. Peak shape functions

There exists a variety of profile functions that can be useful to describe the orientation distribution of preferentially oriented crystallites:

A normalized Gaussian located at ψ_{hkl} with standard deviation σ is mathematically expressed as:

$$G(\psi_{i,j}, \psi_{hkl}, \sigma) = \frac{1}{\sqrt{2\pi}\sigma} \exp\left(-\frac{(\psi_{i,j} - \psi_{hkl})^2}{2\sigma^2}\right) \tag{12}$$

A normalized Lorentzian, mathematically given by

$$L(\psi_{i,j}, \psi_{hkl}, \sigma) = \frac{1}{\pi\sigma} \left(1 + \frac{(\psi_{i,j} - \psi_{hkl})^2}{\sigma^2}\right)^{-1} \tag{13}$$

features slightly steeper flanks and more pronounced peak tails compared to a Gaussian. Through a linear combination of Lorentzian and Gaussian the Pseudo-Voigt function is obtained:

$$V(\psi_{i,j}, \psi_{hkl}, \sigma_L, \sigma_G, \eta) = \eta \cdot L(\psi_{i,j}, \psi_{hkl}, \sigma_L) + (1 - \eta) \cdot G(\psi_{i,j}, \psi_{hkl}, \sigma_G) \tag{14}$$

The factor $\eta \in [0,1]$ describes the fraction of Lorentzian share in the mixed profile. σ_L and σ_G refer to the standard deviation of the Lorentzian and Gaussian, respectively. Finally, the normalized Pearson (VII) function is expressed as

$$S(\psi_{i,j}, \psi_{hkl}, \sigma, \eta) = \frac{1}{\sqrt{\eta}\sigma\, \mathrm{B}\left(\frac{\eta}{2}, \frac{1}{2}\right)} \left(1 + \frac{1}{\eta}\frac{(\psi_{i,j} - \psi_{hkl})^2}{\sigma^2}\right)^{-\frac{\eta+1}{2}} \tag{15}$$

where $\mathrm{B}(x, y)$ is the beta function. The Pearson function is a generalization of the Lorentz function where the weight of intensity between center and tails is adjusted by the parameter $\eta \in [0, \infty)$. It corresponds to a Lorentzian for $\eta = 1$ and to a Gaussian in the limit of $\eta \to \infty$.

S3. X-ray diffraction methods

GIXD measurements were performed at the beamline XRD1, synchrotron Elettra (Trieste, Italy). X-rays with a wavelength of 1.4 Å and a nominal beam size of 200 µm in diameter were used. The anthraquinone and binaphthalene thin films were measured at an incident angle of $\alpha = 1°$. This is well above their critical angles of $\alpha_c = 0.16°$ for anthraquinone and $\alpha_c = 0.15°$ for binaphthalene. In contrast ZIF-8 was measured at an incident angle of $\alpha = 0.15°$, close to its critical angle of $\alpha_c = 0.12°$. For collecting the raw GIXD intensities, a Dectris Pilatus 2M detector was used at a nominal sample-detector distance of 20 cm for anthraquinone and binaphthalene, and 15 cm for ZIF-8. To achieve better statistics, the anthraquinone and binaphthalene diffraction signals were integrated over a full sample rotation around the surface normal, indicated by the rotation angle φ (compare Fig. 2a). In contrast, static measurements without sample rotation were performed for ZIF-8. For calibration a

LaB_6 standard was used. The measured data was transformed into reciprocal space using the software GIDVis (Schrode *et al.*, 2019). Further data processing and evaluation were performed in MATLAB.

S4. Anthraquinone

S4.1. Sample preparation

9,10-anthraquinone was purchased from Tokyo Chemical Industry (TCI) with a purity of >98.0%. Thin films were prepared by dip coating from a 2 g/l tetrahydrofuran solution onto a silicon wafer. For the dip coating process, samples were immersed in the solution and slowly withdrawn at a rate of 1 µm/s for one sample and 2 µm/s for another sample. The used silicon wafers (Siegert Wafers) are atomically flat and native oxide layer terminated. Prior to use, the substrates were cleaned with acetone and isopropanol and sonicated in an isopropanol bath for 10 min. Finally, the substrates were dried in a nitrogen stream. Further details on the preparation of anthraquinone thin films are given in (Gasser, 2022).

S4.2. Experimental results

A total of 12 radial line profiles were extracted from the measured GIXD data of the 1 µm/s and 2 µm/s samples, as shown in Fig. S2 and S3, respectively. In both cases, a uniplanar texture with three different contact planes (001), (100) and (10-2) was assumed, giving the fit parameters shown Tab. S1.

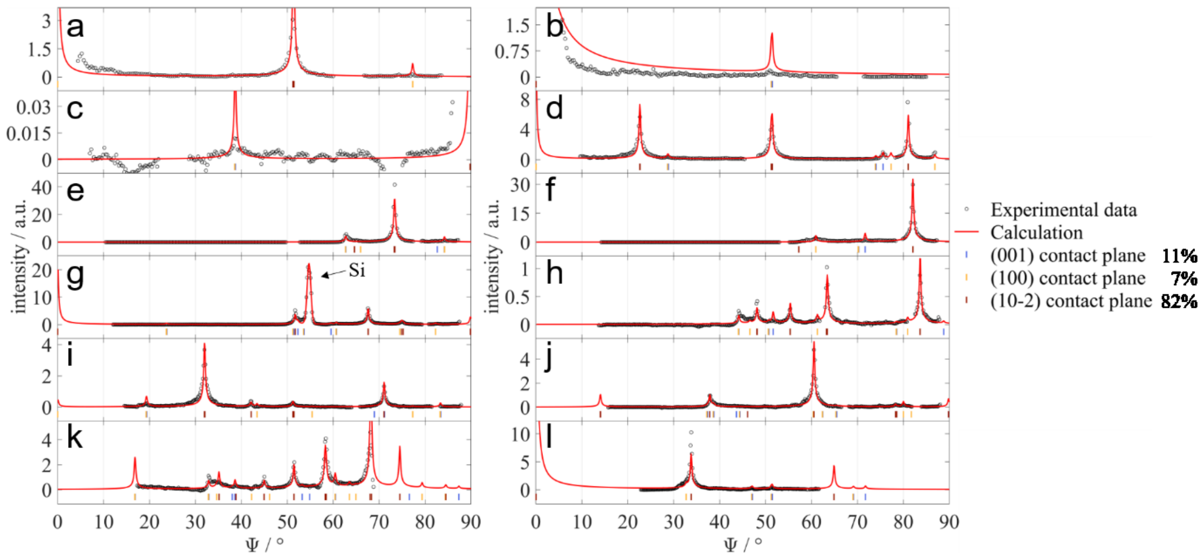

Figure S2 Radial line profiles of an anthraquinone thin film prepared via dip coating using 1 µm/s withdrawal velocity extracted at (a) $q \epsilon$ [0.778,0.858] Å^{-1}, (b) $q \epsilon$ [0.981,1.061] Å^{-1}, (c) $q \epsilon$ [1.236,1.316] Å^{-1}, (d) $q \epsilon$ [1.587,1.697] Å^{-1}, (e) $q \epsilon$ [1.740,1.820] Å^{-1}, (f) $q \epsilon$ [1.830,1.910] Å^{-1}, (g) $q \epsilon$ [1.920,2.120] Å^{-1}, (h) $q \epsilon$ [2.210,2.330] Å^{-1}, (i) $q \epsilon$ [2.340,2.490] Å^{-1}, (j) $q \epsilon$ [2.504,2.664] Å^{-1}, (k) $q \epsilon$ [2.695,2.965] Å^{-1} and (l) $q \epsilon$ [2.975,3.105] Å^{-1}. The results of the fitting algorithm are shown in red. Theoretical Bragg peak positions of the three contact planes are indicated using colored lines below the radial line profiles. The silicon 111 peak was fitted individually and not included in the algorithm.

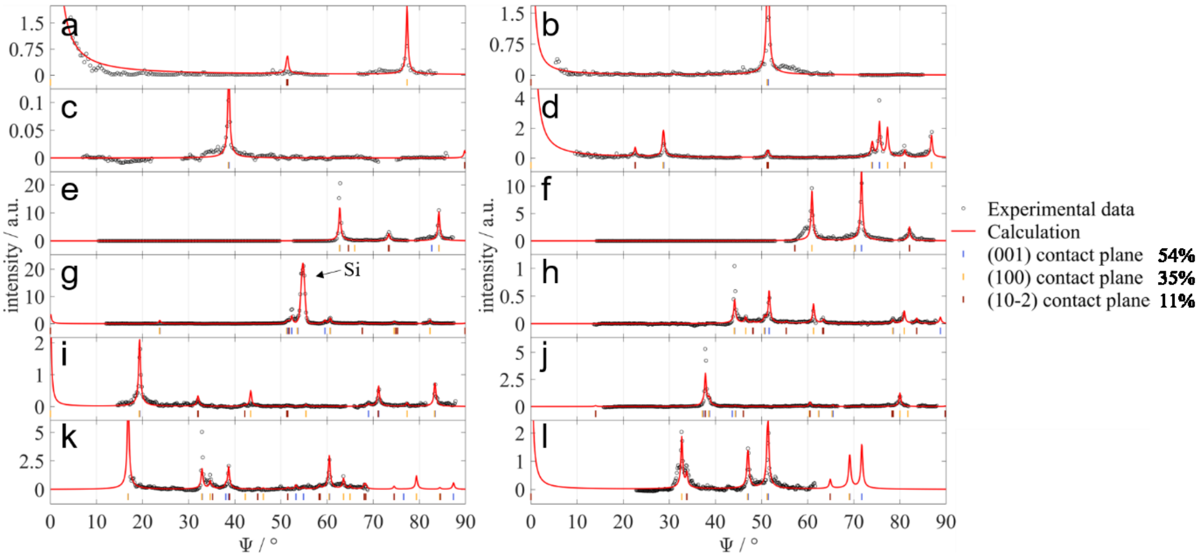

Figure S3 Radial line profiles of an anthraquinone thin film prepared via dip coating using 2 μm/s withdrawal velocity extracted at (a) $q \in [0.778,0.858]$ Å^{-1}, (b) $q \in [0.981,1.061]$ Å^{-1}, (c) $q \in [1.236,1.316]$ Å^{-1}, (d) $q \in [1.587,1.697]$ Å^{-1}, (e) $q \in [1.740,1.820]$ Å^{-1}, (f) $q \in [1.830,1.910]$ Å^{-1}, (g) $q \in [1.920,2.120]$ Å^{-1}, (h) $q \in [2.210,2.330]$ Å^{-1}, (i) $q \in [2.340,2.490]$ Å^{-1}, (j) $q \in [2.504,2.664]$ Å^{-1}, (k) $q \in [2.695,2.965]$ Å^{-1} and (l) $q \in [2.975,3.105]$ Å^{-1}. The results of the fitting algorithm are shown in red. Theoretical Bragg peak positions of the three contact planes are indicated using colored lines below the radial line profiles. The silicon 111 peak was fitted individually and not included in the algorithm.

Table S1 Parameters obtained after fitting of the radial line profiles of anthraquinone thin films prepared via dip coating at withdrawal velocities of 1 μm/s and 2 μm/s. For each contact plane, a scale factor K_{001}, K_{100} and K_{10-2} was obtained. It was assumed, that peak shapes follow a Pearson function with equal standard deviation σ and weight parameter η, independent of the contact plane.

sample	K_{001}	K_{100}	K_{10-2}	σ / rad	η
1 μm/s	0.021	0.014	0.158	0.0072	0.12
2 μm/s	0.045	0.030	0.009	0.0050	0.37

S5. ZIF-8

S5.1. Sample preparation

A silicon wafer was coated with 25 nm of gold on top of a 5 nm Cr adhesion layer using thermal evaporation. After additional cleaning steps, self-assembled monolayer functionalization was achieved by immersing the substrate into a 1 mM octadodecanethiol (ODT) solution in absolute ethanol for 24 h in a non-transparent glass reactor vessel. After functionalization and rinsing, the ZIF-8 thin film was deposited using a two-step molecular layer deposition. A second ZIF-8 thin film was prepared directly on the silicon substrate without functionalization. Further details on sample preparation are given in (Smets *et al.*, 2023, 2025).

S5.2. Experimental results

Three radial line profiles were extracted from the GIXD measurements of each sample as shown in Fig. S4. The radial line profiles were fitted individually, assuming a texture consisting of an oriented and unoriented component, resulting in the fit parameters shown in Tab. S2. Fig. S5 shows the same radial line profiles multiplied with $\sin\psi$. It is particularly interesting to compare the intensities of the 002 and 200 peaks, visualized as red circles in Fig. S1, with the measured radial line profiles in Fig. S4cd and S5cd. Due to the cubic unit cell of ZIF-8, both 002 and 200 peaks share the same structure factor F_{002}. However, due to the uniplanar texture, the out-of-plane 002 peak has a peak multiplicity of $m_{002} = 1$, in contrast to the in-plane 200 peak with $m_{200} = 4$ (since the 200, -200, 020 and 0-20 peaks overlap). Therefore, one might intuitively expect the 200 peak to appear in the radial line profile with 4 times the intensity of the 002 peak. However, this is only achieved after applying the Lorentz correction including the $\sin\psi$-term to the measured data, as shown in Fig. S5cd.

Table S2 Parameters obtained after fitting of the radial line profiles of ZIF-8 thin films prepared on bare silicon and an ODT-functionalized substrate. For each sample, three radial line profiles corresponding to the 110, 200 and 112 Bragg peaks were fitted individually. Therefore, for each radial line profile individual scale factors K_{or} and K_{unor} were obtained for the oriented and unoriented component, respectively. For the oriented fraction, Gaussian shaped peaks were assumed, leading to the standard deviation σ_{or}.

sample	profile	K_{or}	K_{unor}	σ_{or} / rad
Si	110	4.18	4.88	0.22
	002	7.68	6.08	0.22
	112	6.65	6.76	0.21
ODT	110	4.88	0.72	0.12
	002	12.27	0.97	0.13
	112	12.04	1.06	0.12

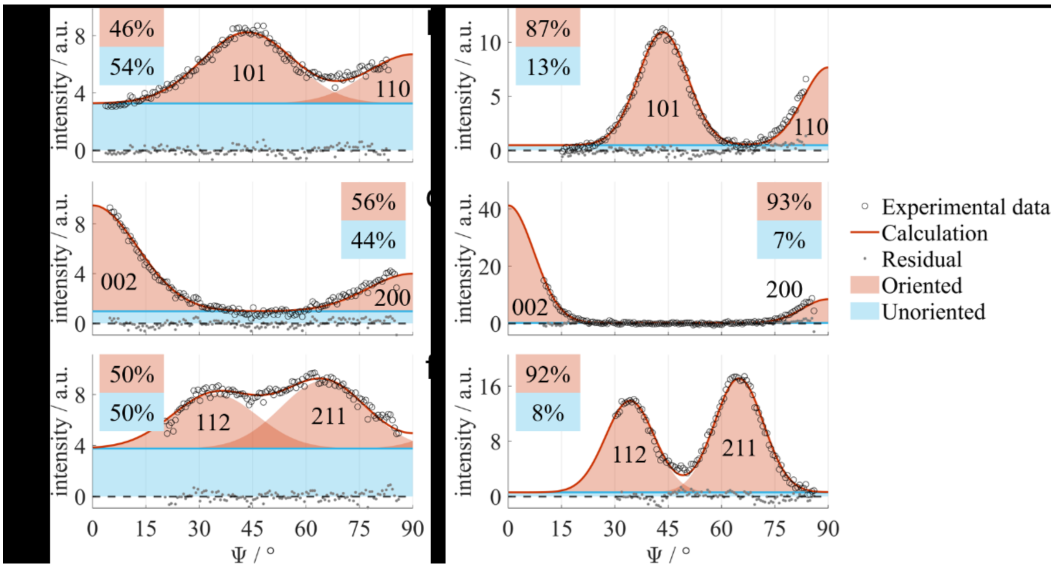

Figure S4 Radial line profiles of ZIF-8 thin films prepared on (a), (c), (e) a bare silicon substrate and (b), (d), (f) an ODT-functionalized substrate. Integration limits were chosen as (a) $q \epsilon$ [0.47,0.57] $Å^{-1}$, (b) $q \epsilon$ [0.485,0.595] $Å^{-1}$, (c) $q \epsilon$ [0.69,0.79] $Å^{-1}$, (d) $q \epsilon$ [0.70,0.82] $Å^{-1}$, (e) $q \epsilon$ [0.84,0.94] $Å^{-1}$ and (f) $q \epsilon$ [0.86,0.98] $Å^{-1}$. The results of the fitting algorithm are shown in red. The red areas correspond to the oriented fraction of ZIF-8, whereas the blue areas correspond to unoriented ZIF-8. Bragg peaks of the oriented fraction are indexed with their Laue indices.

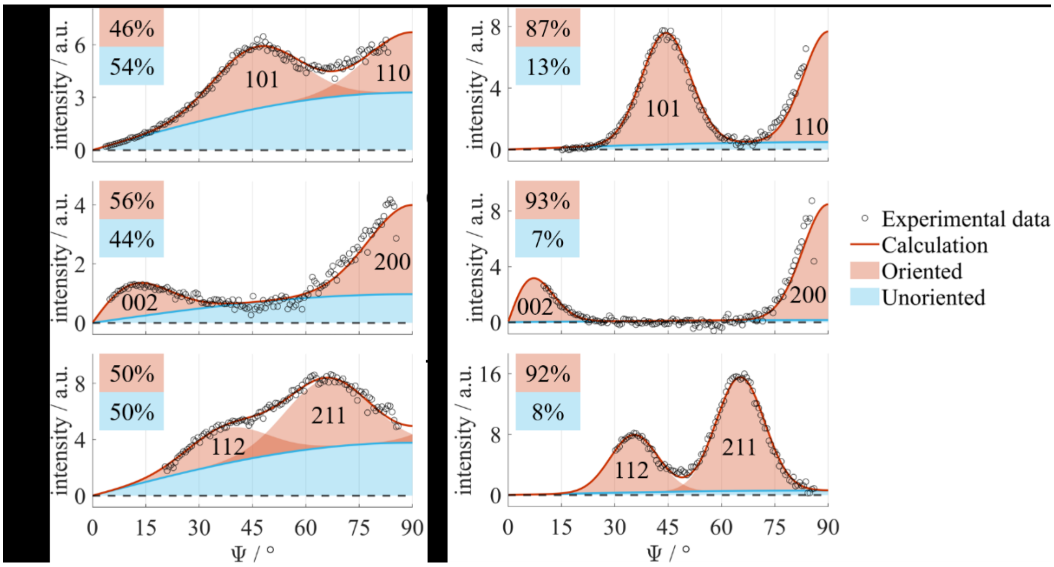

Figure S5 Radial line profiles of ZIF-8 thin films prepared on (a), (c), (e) a bare silicon substrate and (b), (d), (f) an ODT-functionalized substrate corresponding to Fig. S4. Measured and calculated radial line profiles were multiplied with sin ψ so that observed areas are directly proportional to the relative volume fraction of the respective components. Consequently, the red areas are proportional to the amount of oriented ZIF-8, whereas the blue areas are proportional to the volume of unoriented ZIF-8. Bragg peaks of the oriented fraction are indexed with their Laue indices.

S6. Binaphthalene

S6.1. Sample preparation

Racemic 1,1'-binaphthalene (TCI, purity >98) was dissolved in dichloromethane to achieve a 70 g/l solution. Thin films were prepared by spin coating at static dispense and varying spin velocities of 500 rpm, 1000 rpm, 2000 rpm, 4000 rpm and 6000 rpm. Silicon wafers were used as substrates and cleaned following the same protocol as described for the anthraquinone thin films.

S6.2. Experimental results

Two radial line profiles were extracted from the GIXD pattern of each sample, as shown in Fig. S6. The radial line profiles were fitted assuming a film consisting of a racemic phase with uniplanar texture and (100) contact plane and a chiral phase with an unoriented fraction and an oriented fraction with (127) contact plane. The obtained fit parameters are summarized in Tab. S3.

Table S3 Parameters obtained after fitting of the radial line profiles of binaphthalene thin films prepared at varying spin velocities of 500 rpm, 1000 rpm, 2000 rpm, 4000 rpm and 6000 rpm. For each sample, three radial line profiles corresponding to the 110, 200 and 112 Bragg peaks were fitted individually. A scale factor K_{rac}, $K_{\mathrm{ch,or}}$ and $K_{\mathrm{ch,unor}}$ was obtained for the racemic phase of binaphthalene, the oriented fraction of the chiral phase of binaphthalene and the unoriented fraction of the chiral phase, respectively. For the racemic peak, a Pseudo-Voigt peak shape was assumed giving the standard deviations $\sigma_{\mathrm{rac},L}$ for the Lorentzian component and $\sigma_{\mathrm{rac},G}$ for the Gaussian component. η_{rac} defines the Lorentzian share in the Pseudo-Voigt profile. For the oriented fraction of the chiral phase, Gaussian shaped peaks were assumed resulting in the standard deviation $\sigma_{\mathrm{ch,or}}$.

sample	K_{rac}	$K_{\mathrm{ch,or}}$	$K_{\mathrm{ch,unor}}$	$\sigma_{\mathrm{rac},L}$ / rad	$\sigma_{\mathrm{rac},G}$ / rad	η_{rac}	$\sigma_{\mathrm{ch,or}}$ / rad
500 rpm	261.7	28.8	83.2	0.11	0.36	0.43	0.15
1000 rpm	124.9	80.2	175.8	0.08	0.41	0.70	0.15
2000 rpm	30.5	101.0	154.7	0.27	0.08	0.90	0.17
4000 rpm	21.2	131.0	129.9	0.20	0.31	0.39	0.18
6000 rpm	5.2	165.5	83.0	0.22	/	1.00	0.21

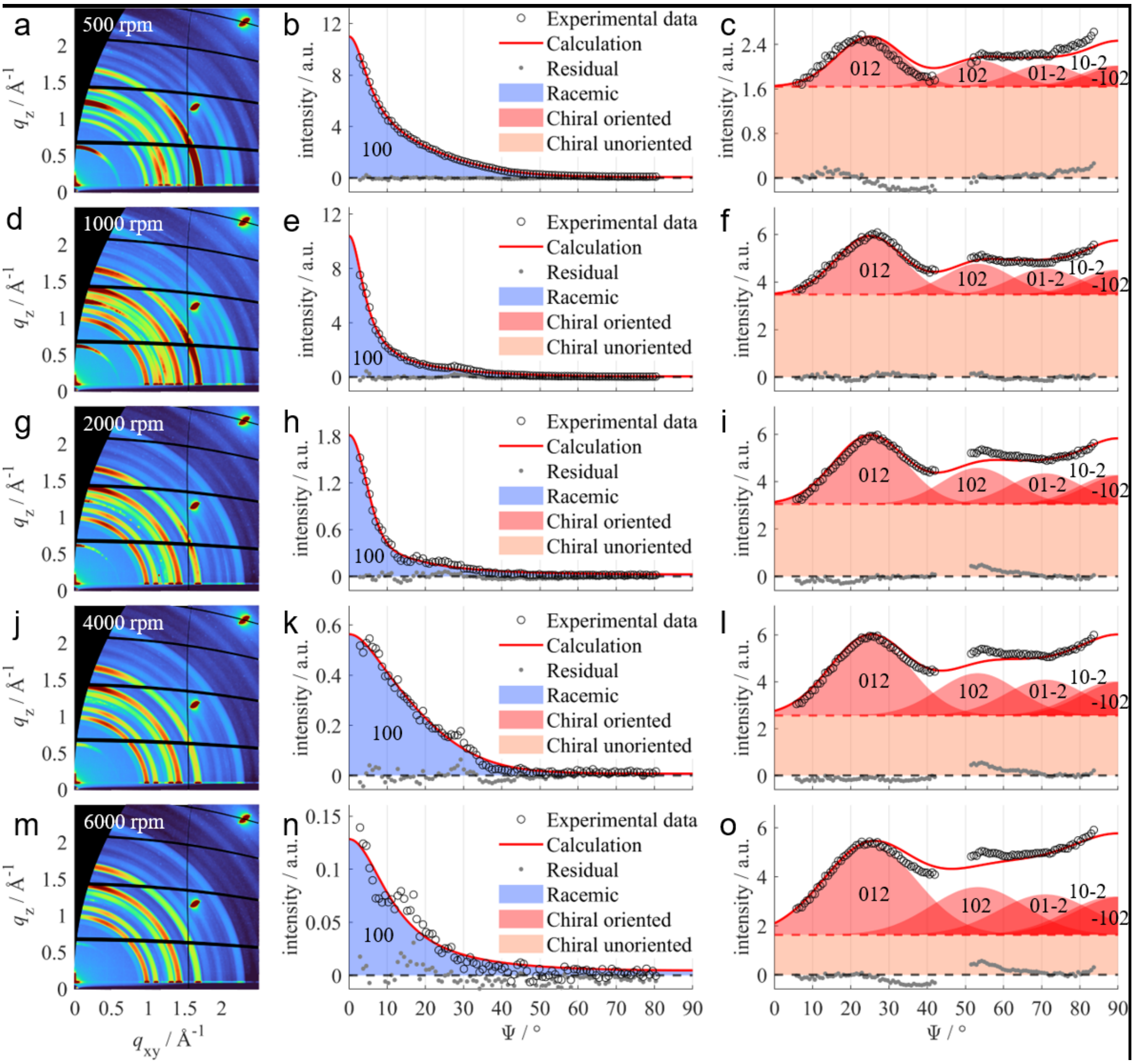

Figure S6 GIXD patterns of binaphthalene thin films prepared via spin coating at varying spin velocities of (a) 500 rpm, (d) 1000 rpm, (g) 2000 rpm, (j) 4000 rpm and (m) 6000 rpm. Radial line profiles were extracted at (b), (e), (h), (k), (n) $q \epsilon$ [0.60,0.63] Å^{-1} and (c), (f), (i), (l), (o) $q \epsilon$ [0.97,1.00] Å^{-1}. The results of the fitting algorithm are shown in red. The blue area corresponds to the racemic phase of binaphthalene, the red area to the oriented fraction of the chiral phase of binaphthalene and the orange area to the unoriented fraction of the chiral phase. Bragg peaks of both oriented components are indexed with their Laue indices.

References

Gasser, F. (2022). Polymorph Screening on Surfaces Applied to Anthraquinone. Master's Thesis, Graz University of Technology.

Smets, J., Cruz, A. J., Rubio-Giménez, V., Tietze, M. L., Kravchenko, D. E., Arnauts, G., Matavž, A., Wauteraerts, N., Tu, M., Marcoen, K., Imaz, I., Maspoch, D., Korytov, M., Vereecken, P. M., De Feyter, S., Hauffman, T. & Ameloot, R. (2023). *Chem. Mater.* **35**, 1684–1690.

Smets, J., Rubio-Giménez, V., Gándara-Loe, J., Adriaenssens, J., Fratschko, M., Gasser, F., Resel, R., Brady-Boyd, A., Ninakanti, R., De Feyter, S., Armini, S. & Ameloot, R. (2025). *Chem. Mater.* **37**, 400–406.
